# Cholinergic Modulation of the Immune System in Neuroinflammatory Diseases

**DOI:** 10.3390/diseases9020029

**Published:** 2021-04-12

**Authors:** Marcella Reale, Erica Costantini

**Affiliations:** 1 Department of Innovative Technologies in Medicine and Dentistry, University “G.d’Annunzio”, 65122 Chieti-Pescara, Italy; 2 Department of Medical, Oral and Biotechnological Science, University “G.d’Annunzio”, 65122 Chieti-Pescara, Italy; erica.costantini@unich.it

**Keywords:** acetylcholine, α7 nAChR, neuroinflammation, cognitive impairment, immune system

## Abstract

Frequent diseases of the CNS, such as Alzheimer’s disease, Parkinson’s disease, multiple sclerosis, and psychiatric disorders (e.g., schizophrenia), elicit a neuroinflammatory response that contributes to the neurodegenerative disease process itself. The immune and nervous systems use the same mediators, receptors, and cells to regulate the immune and nervous systems as well as neuro-immune interactions. In various neurodegenerative diseases, peripheral inflammatory mediators and infiltrating immune cells from the periphery cause exacerbation to current injury in the brain. Acetylcholine (ACh) plays a crucial role in the peripheral and central nervous systems, in fact, other than cells of the CNS, the peripheral immune cells also possess a cholinergic system. The findings on peripheral cholinergic signaling, and the activation of the “cholinergic anti-inflammatory pathway” mediated by ACh binding to α7 nAChR as one of the possible mechanisms for controlling inflammation, have restarted interest in cholinergic-mediated pathological processes and in the new potential therapeutic target for neuro-inflammatory-degenerative diseases. Herein, we focus on recent progress in the modulatory mechanisms of the cholinergic anti-inflammatory pathway in neuroinflammatory diseases.

## 1. Cholinergic System: A Narrow Overview

The term cholinergic system comprises the well-known neurotransmitter acetylcholine (ACh) and the system of synthesizing enzymes, transporters, receptors, and enzymes for degradation. ACh, the first neurotransmitter identified, is synthesized in the cytoplasm from choline and acetyl coenzyme A, in a single-step reaction catalyzed by the enzyme choline acetyl transferase (ChAT). The vesicular acetylcholine transporter (VAChT) uptakes the   neurotransmitter into synaptic vesicles located in axon terminals and is released to the synaptic cleft upon depolarization in a process mediated by an elevation in intracellular Ca^++^ [1,2].

ACh present at the synaptic cleft is hydrolyzed within a few milliseconds into choline and acetate groups by the enzyme acetylcholinesterase (AChE) and butyrylcholinesterase (BChE), forming acetate and choline, which is recycled into the presynaptic nerve terminal by the high-affinity  choline transporter (CHT1). BChE, considered as nonspecific cholinesterase or pseudo-cholinesterase, is a serine hydrolase that catalyzes the hydrolysis of esters of choline. BChE, expressed in distinct populations of neurons, is a co-regulator of cholinergic neurotransmission, and seems to be involved in some aspects of the development of the nervous system [3]. The primary role of AChE is to terminate neuronal transmission and signaling between synapses to prevent ACh dispersal and activation of nearby receptors. The interaction of AChE with the substrate ACh results in the breakdown, hydrolysis, and inactivation of ACh and subsequent control of the amount of ACh at the synapse. AChE is one of the most kinetically efficient enzymes, in fact, a single molecule of AChE can hydrolyze 5000 ACh molecules per second, and a chronic overproduction of AChE can be expected to change ACh balance, inducing activity-dependent secondary feedback responses in the nervous system [4].

Following release, ACh binds briefly two types of receptors: the ionotropic family of nicotinic receptors (nAChR) and the metabotropic family of muscarinic receptors (mAChR). In the central nervous system, nicotinic and muscarinic receptor subtypes are present both on neurons and glial cells where they mediate various physiological effects e.g., motor control, temperature regulation and memory, and synapse function and plasticity. Muscarinic receptors are G-protein coupled receptors subdivided into five subtypes (M1–M5). M2 and M4 are inhibitory receptors, while M1, M3, and M5 are excitatory receptors. Muscarinic receptors are stimulated by muscarine, and showed either excitatory or inhibitory effects, varying on the cell type where these receptors are expressed [5]. Nicotinic receptors may be distinct in two subtypes: α-bungarotoxin-sensitive receptors with homomeric or heteromeric structures made up of the α7-8, and/or α9-10 subunits, and α-bungarotoxin-insensitive receptors with heteromeric structures, composed of the α and β subunits are stimulated by nicotine. From the combination of these different subunits, several different types of nicotinic receptors with distinct properties and functions, can be generated [6]. The mainly characterized nAChR, due to its relationship to immune functions, is α7 nAChR. Activation of α7 nAChR on astrocytes attenuates LPS-mediated upregulation of inflammatory cytokines through inhibition of the NF-κB inflammatory pathway [7], and by phosphorylation of STAT3, a mediator of an anti-apoptotic cascade under inflammatory challenge conditions, playing a key role in neuroprotection [8].

**Figure 1:**
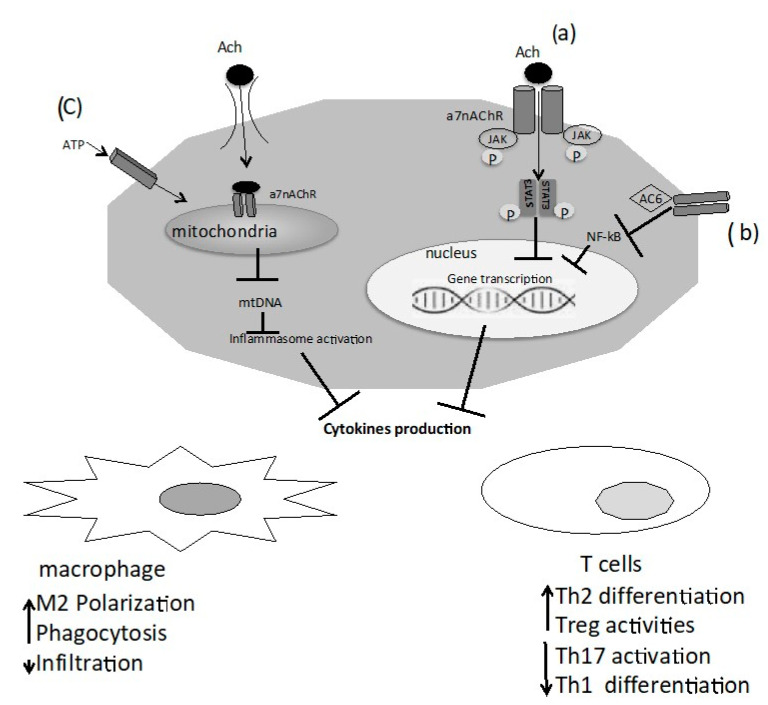
Macrophages and T cells expressed the α7 nicotinic ACh receptor (α7 nAChR), and the binding of acetylcholine (ACh) to α7 nAChR triggers intracellular signaling: (**a**) interaction between α7 nAchR and JAK2 and consequently phosphorylation of STAT 3. Translocation of phosphorylated STAT3 dimers to the nucleus suppresses pro-inflammatory cytokines; (**b**) activation of adenylyl cyclase 6 (AC6) leads to the inhibition of NF-kB activity and suppression of pro-inflammatory cytokines; (**c**) an additional mechanism is the activation by extracellular ATP of immune cells, that run a rapid influx of ACh into the cytoplasm. In the cytoplasm, ACh interacts with mitochondrial α7 nAChR and reduces mitochondrial DNA release, and inhibits inflammasome activation and cytokine release.

The functioning of nAChRs was believed to be related exclusively to the plasma membrane, but several studies reported that mitochondria express α3β2, α4β2, α7β2, α9 nAChR subtypes. α7 nAChRs, the most abundant nAChR subunit in the mitochondria, are expressed at the outer membrane and have certain connections with a marker of the outer membrane, voltage-dependent anion channels (VDACs) and could mediate the exchange between the cytosol and the intermembrane space of the mitochondria regulating the release of pro-apoptotic factors, such as cytochrome C [9,10]. Lykhmus et al. showed that the nAChRs found in the brain mitochondria are modulated by inflammation and are involved in the accumulation of amyloid peptides, and apoptosis [11]. ACh and cholinergic agonists attenuate ATP-induced mitochondrial perturbation and inhibit NLRP3 inflammasome activation by preventing mitochondrial DNA release and subsequent inhibition of inflammasome-dependent inflammatory cytokines such as IL-1β, IL-18, and HMGB1 [12] (Figure 1).

### Cholinergic System in Immune Cells

Kawashima and Fujii [13] described the presence of an extraneuronal cholinergic system in lymphocytes isolated from thymus, lymph node, spleen, and peripheral blood. Many immune cells have been demonstrated to possess essential components of the cholinergic system (Table 1), and the expression of both muscarinic and nicotinic ACh receptors in lymphocytes and thymocytes was confirmed in binding studies using radiolabeled mAChR and nAChR ligands. The considerable levels of ACh present in the mammalian blood led to the hypothesis that ACh was produced also by non-neuronal cells, as confirmed by a specific radioimmunoassay that detected ACh in peripheral blood cells. T cells are the main source of the ACh present in the blood. In fact, in T lymphocytes, the ACh level was three times higher than in B cells, and CD4^+^ T lymphocytes contained more ACh than CD8^+^ cells [14]. This non-neuronal ACh likely functions as a local signaling molecule and may regulate basic cell functions. In non-neuronal cells, ACh is involved in cell proliferation and differentiation, cytoskeletal organization, and cell–cell contact, and it is highlighted that the ACh produced by the inflammatory cells has effects on those cells, suggesting that ACh modulates the activity of inflammatory cells via autocrine and paracrine loops by the binding to the α7 nicotinic receptor. In human mononuclear cells, the synthesis of ACh from acetyl coenzyme A and choline is regulated with a positive feedback mechanism through the transcription of ACh synthetic enzyme, choline acetyltransferase (ChAT), detected for the first time by Fujii et. colleagues [15]. The presence of ChAT mRNA and protein has been detected in un-stimulated human CD4^+^, but not CD8^+^ T cells or B cells, in accord also with ACh level detected in these cells. In T cells, PHA activates protein kinase C (PKC) and mitogen-activated protein kinase (MAPK) pathways via the T-cell receptor (TCR)/CD3 complex, induces the expression and activity of ChAT, and increases the synthesis of ACh, which was involved in the induction of T-cell subset maturation and activities [16]. Human peripheral blood T lymphocytes, but not B lymphocytes, possess high degrading enzymes AChE and BChE. The AChE enzyme was shown to be membrane-bound and present in homogenous dimeric form, and a significant increase in AChE activity was observed as an early response to various stimuli in normal human peripheral blood lymphocytes [17,18]. More recently, expression of three different types of the AChE mRNAs has been detected in human peripheral blood lymphocytes [19] and changes in lymphocyte AChE activity were related to cell dysfunction reflecting immune deficiency. AChE shares an approximately 54% amino acid sequence identity with the BChE that, with a lower conversion rate of Ach with respect to AChE, plays a greater role in the blood than in the nervous system.

The effects of cholinergic agonists on leukocyte proliferation, cytotoxic activity, and on the number of antibody-producing cells have pointed out the presence of mAChRs or nAChRs in lymphocytes [16] as confirmed by a ligand binding and reverse transcription-polymerase chain reaction (RT-PCR) assay [13], supporting the suggestions that the cholinergic system is involved in the regulation of immune function.

Sato et al. showed that different combinations of mAChR subtypes could be expressed in lymphocytes of each individual subject. In fact, the expression of mRNAs for the M4 and M5 receptor subtypes was constant, while the expression of mRNAs encoding the M1, M2, and M3 subtypes was variable [21] in relationship with individual immunological status. The expression patterns and intensities of mAChRs were modulated by anti-CD3 and anti-CD28, stimulation of lymphocytes with PMA plus ionomycin leads the M5 mAChR gene expression and the activation of M1 and M2 muscarinic receptor by ACh, enhances interleukin-2 (IL-2) production, suggesting a role for mAChRs in the regulation of immune function. Kawashima and Fujii suggested that the expression pattern of mAChRs may be genetically determined or controlled by factors such as infection and physiological stress [13].

Through the use of specific nAChR agonists, which induce changes in the intracellular concentration of Ca2^+^, the presence of functional α2, α5, α6, α7, α9, and α10 nAChR subtypes in both T and B lymphocytes has been demonstrated [13]. The levels and roles of nicotinic receptors of α4(α5)β2 and α7(α5β4) subtypes expressed in B-lymphocytes changed during the cell maturation steps. In fact, in the bone marrow, B-lymphocytes respond to ACh produced by cholinergic nerve terminals, while circulating B cells respond to ACh produced by activated T lymphocytes [13]. Expression of nicotinic receptors reinforces B-lymphocyte survival during differentiation/selection but inhibits their transition to mature state and proliferation, affecting expression and/or signaling of CD40. In mature B lymphocytes, the nicotinic receptor deficiency influences the percentage of cells, which switched from IgM to IgG production [22]. β2 nicotinic receptor deficiency affected B lymphocyte (CD40), but not T lymphocyte (CD3) proliferation, and the ACh, endogenously produced by T lymphocytes interacting with nicotinic receptors, may represent an additional pathway for the cross talk of B and T lymphocytes [23].

The activation of α7 nAChRs suppresses the differentiation of T cells into Th1 and Th17, enhances differentiation into Th2 cells of naïve CD4^+^ T cells, and increases numbers of Tregs among CD4^+^ CD62L^+^ T cells, non-specifically activated using anti-CD3/CD28 Abs, most likely via activation of JAK2/STAT pathways [24]. Secreted Ly-6/uPAR-related protein 1 (SLURP-1), the endogenous allosteric and/or orthosteric α7 nAChR ligand, is expressed in peripheral blood MNLs, DCs, and Mϕs, and its crucial role in the T cell activation elicited by immunological stimulation was suggested. SLURP-1, together with ACh synthesized by CD4^+^ T cells, modulates immune function and inflammatory responses acting via ACh receptors on immune cells [25].

The presence of a “cholinergic anti-inflammatory pathway” proposed by Borovikova et al. [7] suggests that ACh, released in response to vagal activation, influenced local inflammation reducing the release of pro-inflammatory cytokines. Efferent vagus fibers respond to cytokine inflammatory input releasing ACh that activate α7 nAChR and initiate the anti-inflammatory response.

Recently Rosas-Ballina and colleagues found that in the cholinergic anti-inflammatory pathway, the ACh was not of neural origin, but T lymphocytes are able to synthesize ACh that, by binding to α7, nAChR on macrophages inhibits the nuclear translocation of NF-kB, and intercepts the production of pro-inflammatory mediators [26]. The anti-inflammatory role of α7 nAChR has been highlighted by observing the stimulation of enteric macrophages through the activity of the vagal nerve [26], in which the activation of α7 nAChR induces the activation of Janus kinase-2 (JAK-2) and the subsequent phosphorylation of the signal transducer and activator of transcription-3 (STAT-3) which dimerizes and translocates to the nucleus by inhibiting the gene expression of pro-inflammatory cytokines such as tumor necrosis factor-α (TNF-α) and interleukin-6 (IL-6) [27]. Further confirmation of the anti-inflammatory activity of α7 nAChR was obtained with the use of specific α7 receptor antagonists, CHRNA7 knock-out mice [28], or overexpression of its duplicate dominant negative form dupa7 expressed only in humans [29]. Distinct nicotinic receptors regulate the production of pro-inflammatory and anti-inflammatory cytokines, and the activation of α bungarotoxin-sensitive α7 receptors inhibit the expression of TNF-α both at the mRNA and protein level, whereas activation of mecamylamine-sensitive receptor subtypes controls the production of IL-6 and IL-18 at the protein level [30].

The discovery of T-lymphocyte cytotoxicity via the muscarinic cholinergic system exposed the cholinergic anti-inflammatory pathway [31] and was reinforced by the inhibitory effects of nicotine on T-cell differentiation and responses [32]. Nicotine, influencing nAChR, is more active than ACh in inhibiting the production of pro-inflammatory cytokines by macrophages, but its therapeutic benefit is limited due to troublesome side-effects.

## 2. Cholinergic and Immune System Crosstalk in Widespread Neuro-Inflammatory Diseases

Neurodegenerative diseases are a group of chronic, progressive disorders characterized by the gradual loss of neurons, and specific portions of the brain, spinal cord, or peripheral nerves are affected, with consequent impairment of cognition, movement, strength, coordination, sensation, or autonomic control. In the pathogenesis of several neurodegenerative diseases such as Alzheimer’s disease (AD), Parkinson’s disease (PD), and multiple sclerosis (MS), although it may not typically represent an initiating factor, inflammation is associated with degeneration of the nervous system, and is characterized by the activation of microglia and astrocytes, secretion of pro-inflammatory cytokines and chemokines, and the recruitment of more immune cells from the periphery. Under physiological conditions, microglia exhibit a deactivated phenotype that, in the presence of pathogen or tissue damage, switch to an activated phenotype and thereby promote an inflammatory response. Classically activated microglia or “M1” release inflammatory mediators, including proinflammatory cytokines, free radicals, and complement, help microglia to communicate with astrocytes and peripheral immune cells. Chronic and excessive microglial activation leads to the pro-inflammatory and neurotoxic environment and contributes to neurodegeneration and cognitive dysfunction [33,34,35,36,37]. Microglia induced by Th2 type cytokines such as IL-4, IL-10, or IL-13, or alternatively induced M2, produced anti-inflammatory molecules and neurotrophic factors implicated in repairing/remodeling and neuroprotective effects on endangered neurons [38,39]. Also, astrocytes, a significant component of the blood-brain barrier, behave as effective immune cells in the CNS playing a dual role, in fact, they can both amplify the effects of inflammation and mediate cell damage and protect the CNS. Similar to microglia, astrocyte polarization into the pro-inflammatory A1 and anti-inflammatory A2 phenotype, which is similar to M1 and M2 polarization of microglia, has been described in vitro and in vivo. Activated astrocytes are endowed, as microglia, with the ability to secrete cytokines and chemokines, which exert an impact on both adaptive and innate immune responses. As a prominent source of cytokines, chemokines, and other inflammatory mediators, astrocytes are instrumental both in the propagation as well as in the down-regulation of inflammatory responses in the brain. Due to their strategic location at the blood–brain interface, they are important for blood–brain barrier function and in particular in controlling the spread of inflammatory cells from the perivascular space into the parenchyma [40]. Neurons, releasing neuropeptides and neurotransmitters, regulate inflammation and favor the differentiation of T-regulatory cells. On the other hand, Tregs infiltrate the brain and show an activated/memory phenotype, suggesting that T cells are activated peripherally and then reactivated in the CNS [41]. The involvement of adaptive cellular immune responses in neurodegenerative disorders has been deduced from detections of T-cell responses to specific CNS antigens, or shifts in CD4^+^ and CD8^+^ cell populations in the periphery as well as in the CNS [42]. T cells have been implicated in complex brain processes including spatial learning, memory, emotional behavior, and stress responsiveness. CD4^+^ T cells are recruited to the meninges and secrete interleukin-4 that skews macrophages and microglia to an M2 anti-inflammatory phenotype and induces the production of brain-derived neurotrophic factor by astrocytes, leading to the improvement of spatial learning and memory [43,44,45,46,47]. B cells and CD4^+^ and CD8^+^ T cells, can play a role in neurodegeneration which is also evidenced by the close association of T cells expressing TNF-related apoptosis-inducing ligand (TRAIL) with dying spinal motor neurons in MS, and alterations in peripheral levels of CD4^+^ and CD8^+^ T cells, as observed in AD [48,49].

**Figure 2:**
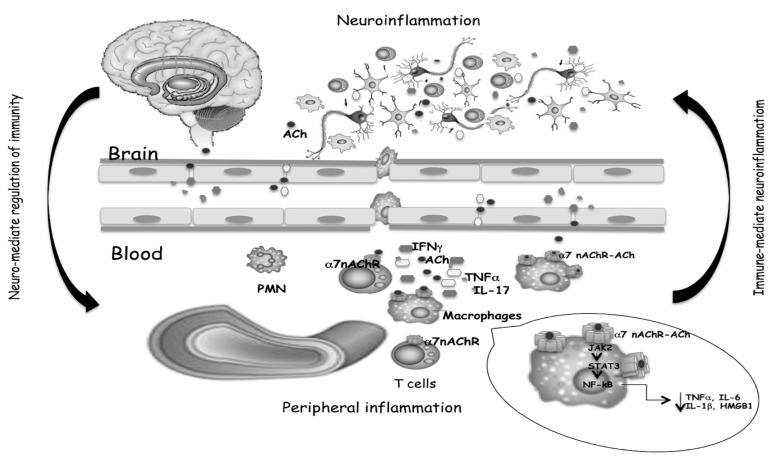
Cytokines and neurotransmitters mediate the bidirectional crosstalk between nervous and immune systems. The binding of ACh, produced by T cells, to α7 nAChR expressed on macrophages, triggers inhibitory signals that limit inflammatory cytokine release, as well as T cell activation and differentiation. Both the reduced production of peripheral cytokines and the reduced cell migration from the periphery to the brain, help to mitigate neuroinflammation.

Consequently, the activation of T cells in CNS, increased levels of the inflammatory cytokines TNF-α and IL-6, and the chemokine CXCL8 are detected in many neurodegenerative disorders. The same cytokines circulating in the blood can communicate with the brain or crossing the intact blood–brain barrier or spreading easily from the blood into the brain parenchyma [50]. In the brain, cytokines may activate endothelial cells, and in turn activate adjacent perivascular macrophages, which then communicate with microglia. The reported correlation between increased cytokine levels in cerebrospinal fluid (CSF) and plasma and neurodegeneration [51,52,53,54] indicates that the same mediators of immune responses are present in the brain and periphery, and a link between systemic inflammation and neurodegeneration was corroborated by migration from the periphery to the brain of inflammatory cells [55], confirming the communication between the brain and peripheral immune systems (Figure 2). Systemic inflammation with the secretion of pro-inflammatory cytokines in the periphery has been evidenced in MS and AD. Circulating monocytes can migrate in the brain, and together with resident microglial cells, respond to inflammatory stimuli and increase the immune response [56] playing a key role in neurodegenerative disease progression. Th1 and Th17 CD4^+^ T cells infiltrate both the white and gray matter of MS or AD patients, producing inflammatory mediators involved in neuronal loss, that positively correlate with the disease course [57].

On the other hand, it is not surprising that receptors for neurotransmitters expressed in the nervous system may be also expressed on the surface of immune system cells. Some subtypes of glutamate receptors (GluRs), ACh-receptors, serotonin receptors (5-HTRs), dopamine receptors (DARs), and adrenergic receptors are expressed on immune cells [58]. Neurotransmitter-mediated modulation may drive polarization of the peripheral T cell response toward Th1, Th17, or Tregs phenotype. Peripheral immune cells have their own cholinergic system able to release ACh which may act as a modulator of inflammation by binding to α7 nAChR on macrophages, and consequent inhibition of the nuclear translocation of NF-kB, phosphorylation of JAK2, STAT3 translocation into the nucleus and block of pro-inflammatory mediators production [59]. α7 nAChR-dependent cholinergic signaling, in addition to “early” inhibition of pro-inflammatory cytokines production, suppresses the HMGB1, a “late” mediator of systemic inflammation [60].

Peripheral decreases in ACh would also increase concentration and potentiate the effects of pro-inflammatory cytokines within the brain. AChE levels participate in the regulation of peripheral pro-inflammatory cytokine production, which can penetrate the brain and modify neuronal functioning. In fact, AChE hydrolyzes Ach, reducing its levels, and consequently activates α7 nAChR and the anti-inflammatory route. The ‘‘cholinergic hypothesis’’ assumes that impaired cholinergic function has a strategic relevance in neurodegenerative processes. In fact, previous studies have shown that cholinergic system dysfunction caused by dis-regulated ACh synthesis or hydrolysis and accompanied by an altered response of the cholinergic receptors, both of nicotinic and muscarinic types, plays a strategic role in neurodegenerative disease onset and progression.

In light of this, the CNS is no longer considered an “immune-privileged site” but a “special immune-controlled site” and cytokines, neurotransmitters, and trophic factors are mediators of the bidirectional neuro-immune interactions, crosstalk, and positive-feedback loops between immune cells and the central, peripheral, sympathetic, parasympathetic, and enteric nervous systems. The imbalance of this extensive communication may support the onset of immune disorders and may represent an important component of the pathogenic mechanisms involved in neurodegenerative diseases.

### 2.1. Multiple Sclerosis

Multiple sclerosis (MS) is one of the most common chronic inflammatory diseases of the CNS [61,62]. Traditionally, MS has been considered to be an autoimmune disease in which the interruption of the blood–brain barrier integrity, migration, and infiltration of activated lymphocytes, macrophages, and dendritic cells into the central nervous system (CNS) resulting in demyelination, neuro-axonal degeneration, and neurological deficits. Alternatively, it has been suggested that the formation of the oligodendrocyte–myelin complex that drives cyto-degeneration is the early event followed by the release of antigenic debris that induces an autoimmune and inflammatory response [63]. The impaired balance of Th17 cells and Tregs cells is involved in MS, in fact, Th17 cells produce pro-inflammatory cytokines while Treg mediated the production of cytokines that are involved in the maintenance of immune homeostasis and in the control of MS progression, and the Treg/Th17 ratio was negatively correlated with the severity of symptoms [64]. Th1 and Th17 cells specific for myelin oligodendrocyte glycoprotein (MOG) increase microglial activation and drive inflammation, increasing cytokine release [65], confirming that the interaction between peripheral immune cells and microglia was complex and often opposing. Several experimental models have strengthened that nicotine has an anti-inflammatory effect by preventing the polarization to Th1/Th17 [66], that nicotine upregulates the expression of cytotoxic T-lymphocyte-associated antigen 4 (CTLA-4) and Foxp3 and boosts the recruitment of Treg cells to the central nervous system CNS [67,68], and that α7 nAChR improves the differentiation of Treg cells.

The involvement of the cholinergic system in MS is not well-known, but studies on EAE mice have suggested how the treatment with cholinesterase inhibitors may recover motor and cognitive impairment and downgrade the neuro-inflammation. In addition, a role for the cholinergic system was strengthened by the observation that α7 nAChR activation can lead to the inhibition of lymphocyte proliferation [69,70] and to the inhibition of macrophage and microglia activation [71,72]. Stimulation of α7 nAChR on endothelial cells additionally controls the extravasation of leukocytes during inflammation [73], although it is not known if this may happen in the blood–brain barrier. Inflammatory cytokine production in peripheral blood cells was suppressed by cholinergic agonists, as reported by in vitro studies carried out to test the effects of cholinergic agonists on cytokine production. It has been reported that in stimulated human monocyte-derived macrophages Ach, choline, nicotine, and other agonists inhibited pro-inflammatory cytokine release through the α7 nAChR dependent mechanism [72,74,75,76]. Activation of α7 nAChR in PHA-induced PBMC from MS patients by nicotine reduced the production of both inflammatory cytokines IL-17 and IL-1β [77]. Stimulation of CD4^+^ cells by nicotine increased expression and activation of α7 nAChR and reduced the Th17 response [78].

The involvement of the cholinergic system in MS was confirmed also by the detection of lower ACh levels in serum and CSF of MS patients compared with healthy subjects, and increased activity of the ACh hydrolyzing enzymes AChE and BChE and their expression levels in peripheral blood mononuclear cells. In MS patients, the expression of transcript for OCTN-1 and mediatophore, the two proteins typically expressed in immune cells and responsible for the non-vesicular ACh release, were also up-regulated, other than the ACh biosynthetic enzyme ChAT [79,80]. The dysregulated balance between ACh, AChE, and BChE could be responsible for higher pro-inflammatory cytokines production detected in MS patients [81].

The study of Jiang et al. observed that in MS patients the higher ACh content in NK cells was associated with an increased expanded disability status scale (EDSS) score other than with the higher number and volume of lesions, though ACh-producing NK cells might modulate infiltration of monocytes/macrophages and attenuate CNS inflammation [82]. In MS patients, it is likely that inflammation related to disease severity may induce NK cells to release lower ACh or express higher ChAT levels. Even NK cells produced ACh, which was degraded by enhanced production of AChE, contributing to disease severity. In fact, elevated serum levels of ACh in MS patients correlate with better disease outcomes.

Thus, taking into account that in immune cells of MS patients showed increased activity of the cholinergic hydrolyzing enzymes BChE and AChE, reduced levels of ACh, increased pro-inflammatory cytokines production, and a correlation between the genetic polymorphisms and activity of BChE and AChE hydrolyzing enzymes, understanding the role of cholinergic components in the aberrant immune response and severe neuro-inflammation may help the development of new treatments to ameliorate the clinical symptoms and delay or arrest the onset of the disabilities in MS.

### 2.2. Alzheimer’s Disease

Alzheimer’s disease (AD) is a progressive neurodegenerative disease and its neuropathological hallmarks are neuritic amyloid plaques derived from misfolded fragments of amyloid beta (Aβ) that aggregate to form oligomers, fibrils, and insoluble plaques, and neurofibrillary tangles originate from deposits of hyperphosphorylated degenerate filaments, which result from aggregations of the microtubular protein tau. The presence of neuronal plaques activates inflammatory responses mediated by astrocytes and microglia with resulting activation of the immune response and production of cytokines by macrophages and neutrophils, causing damage to neurons [83]. Aβ oligomers affected cholinergic synapses and the synaptic loss is correlated to cognitive impairment. ACh has an important role in cognitive processes, thus the cholinergic system is potentially an important factor in many forms of dementia, including AD, where ChAT transcription and activity is diminished in accordance with the progression of dementia and AChE interacts with the Aβ peptide and stimulates amyloid fibril formation [84]. In this context, AChE inhibitors (ChE-Is) are currently the most frequent treatment for AD patients. Autoradiography assay showed that, in the hippocampus of transgenic Tg2576 mice used as a model of AD, the choline uptake and the expression of muscarinic and nicotinic cholinergic receptors were reduced [85]. The inflammatory reaction to Aβ involves the release of a pro-inflammatory cytokine IL-1β, activated via cleavage by caspase-1 in the inflammasome. The activation of the anti-inflammatory cholinergic pathway through the α7 nAChR, inhibits the activation of the inflammasome [12].

As secondary components in senile plaques, cytokines, chemokines, complement components, and acute-phase proteins are over-produced or co-localized in AD brains [86]. Increased expression of IL-1β and TNFα in the olfactory bulb of the APP SW Tg2576 mouse was related to the accumulation of Aβ and to the increase in ChE and nAChR expression [87]. Increased levels of circulating pro-inflammatory cytokines, such as IL-1α, IL-1β, IL-6, and TNF-α, complement and the presence of activated microglia, have been described in patients with AD, and a direct correlation has been established between Aβ-induced neurotoxicity and cytokine production, and between AD progression, rapid cognitive decline and peripheral immunity and inflammation [88]. In fact, pro-inflammatory cytokines may induce the synthesis of Aβ that, by a positive feedback mechanism, may induce the expression of these cytokines in astrocytes and microglia, and the concept that AD, a disease affecting the central nervous system, can be considered a systemic disorder becomes more and more consistent [89].

A relationship between peripheral inflammatory mediators, Aβ levels, and ApoE genotype was evidenced in Alzheimer’s disease patients [90]. T cells may play a key role in AD-associated inflammation, and an higher percentage of T cells with a more marked response to β-amyloid were shown in AD patients, than healthy subjects. In the brain of AD mice, facilitated by increased BBB permeability, an increase of CD4^+^ and CD8^+^ cells linked to gliosis and amyloid pathology has been observed [91], and Th1 and Th17 cells induced microglial activation modulating phagocytic and secretory phenotype, while Th2 cells had no effect on microglial cytokine production [92].

AChE inhibitors, the most used drugs of AD treatment to slow neurodegeneration, induce a synaptic increase in ACh and a direct stimulation of ACh receptors, and may affect the inflammatory activity of cells participating in AD-associated inflammation, inducing a Th1 to Th2 switch [93] and decreasing inflammation by a dose-dependent reduction in inflammatory cytokine levels [94,95]. Since mAChRs can modulate cognition and ACh via mAChRs expressed on leukocytes may potentiate inflammatory reactions, these receptors could represent a significant therapeutic target to ameliorate the behavioral and cognitive deficits and to regulate immune cell activation in inflammatory conditions of several diseases.

The cholinergic anti-inflammatory pathway represents the efferent/motor arm of the inflammatory reflex, which is a neural circuit that controls the immune response against injuries. Thus, immunological reactions that take part in the immune response of AD patients may be influenced by the modulation of the non-neurological cholinergic system. Knowledge of the non-neuronal cholinergic pathways can prove invaluable in understanding how immunomodulation in AD can represent an important therapeutic target for the development of therapies capable of preventing or halting the development of neuroinflammation and cognitive decline.

### 2.3. Parkinson’s Disease

Although Parkinson’s disease (PD) is a complex and multisystem disorder, neuroinflammation contributes to disease progression through loss of dopaminergic (DA) neurons in the substantia nigra (SN) of the brain. Loss of cholinergic neurons, reductions of ChAT, evidenced cholinergic deficits in PD with potential relevance for symptoms including cognitive and attentional impairments. Microglia activated by α-synuclein, have the potential to produce several neurotrophic factors, apoptosis-related proteins, and many pro-inflammatory cytokines, via the transcription factor NF-κB, exacerbating the progression of PD [96]. Notably, microglia are enriched in SN compared to other regions of the brain. These findings, coupled to reduced antioxidant capacity and enhanced sensitivity of neurons to pro-inflammatory molecules, support a role for microglia-mediated DA degeneration in PD [97]. Other than microglia activation, the neuroinflammation in PD is characterized by astrocyte activation and blood–brain barrier damage. The study of Brochard et al. showed that CD4 and CD8 T lymphocytes are infiltrated in the substantia nigra, indicating that in addition to central glial activation, peripheral T lymphocytes are involved in the neuroinflammation during the pathogenesis of PD [98]. These central/peripheral immune cells drive the production of many pro-inflammatory cytokines to amplify inflammatory signals, and neurotoxins that act on dopamine neurons inducing their death.

In summary, during the course of PD the development of immune inflammation, which is characterized by glial cell activation, peripheral immune cell infiltration, immune complex deposition, and the production and release of many pro-inflammatory cytokines and chemokines, was observed [99]. These findings suggest that Parkinson’s had an important inflammatory contribution, and this inflammation could be related to the cholinergic system [100].

Increasing evidence connect mitochondrial perturbation with neuronal diseases, such as AD and PD [101,102]. In addition to the expression on the cell surface, α7 nAchR is expressed also in the mitochondrial outer membrane in non-neuronal cells, including monocytes and B lymphocytes, and α7 nAchR signaling attenuates ATP-induced mitochondrial perturbation, and ACh inhibits ATP-induced NLRP3 inflammasome activation by preventing mitochondrial DNA release into the cytoplasm, representing the new anti-inflammatory targets. Additionally, the pro-inflammatory responses may be modulated by activation of α7 in macrophages via activation of the Jak2/STAT3 signaling cascade [103], and the upregulation of heme oxygenase-1 (HO-1) may be an alternative mechanism for the cholinergic regulation of pro-inflammatory responses [104]. Thus, activation of this non-neuronal cholinergic system may underlie neuroprotection through the effect of α7 nAchR agonists or AChE inhibitors that improve ACh levels [105].

## 3. CholinomiRs: A Novel Approach for Neuroinflammatory Diseases

MicroRNAs (miRNAs) are non-coding transcripts of 18–25 nucleotides and usually target mRNAs to influence post-transcriptional gene expression affecting the mRNA stability and/or translational repression of their target mRNAs. MiRNAs are involved in pathophysiological processes such as differentiation, proliferation, apoptosis, metabolism, the fate of neuronal and immune cells, and the regulation of the cholinergic system [106,107,108]. Over 200 miRNAs, which modulate both neuronal and immune processes, are identified to target different cholinesterase transcripts and have been designated by Nadorp and Soreq “CholinomiRs” [109]. Simulating the activation of α7 nAChR or influencing cholinesterase activity, miRNA could be helpful for inflammatory and neurodegenerative diseases and represent attractive targets for designing therapies aimed to restore the cholinergic anti-inflammatory pathway also in neuroinflammatory conditions [110].

A large number of miRNAs, targeting ChAT, VAChT, AChE-S, AChE-R, and BChE, can regulate several biological pathways, by the increase of AChE synthesis and decrease of ChAT production that modulate ACh levels.

Nardop and Soreq suggested the overlapping miRNA regulation as a new surveillance mechanism that can balance cholinergic neurotransmission and may be of value for both basic and translational aspects of neuroinflammation-related disorders [109] and showed that few overlapping miRs for BChE and AChE were detected, suggesting that these enzymes are subject to distinct modes of miR regulation.

In the central nervous system miR-124 acts as a controller of microglia quiescence, but also as a modulator of monocyte and macrophage activation in the periphery. In experimental autoimmune encephalomyelitis (EAE), miR-124 was down-regulated in activated microglia, and the conversion of activated macrophages to an inactive phenotype such as resting microglia was linked to the overexpression of miR-124. Peripheral administration of miR-124 reduced CNS inflammation and suppressed EAE by inhibiting macrophages, myelin-specific T cells, and migration of encephalitogenic T cells from the periphery to the site of inflammation [111]. miR-124 driving macrophage polarization from an M1 toward an M2 phenotype may modulate the inflammatory diseases associated with macrophage activation in EAE. Activation of α7 nAChR up-regulates miR-124 expression in LPS-stimulated macrophages reducing pro-inflammatory cytokine production, and in turn, miR-124 targeting STAT3, decreases STAT3 and its phosphorylation and reduces IL-6 transcription, or miR-124 targeting TACE modulates TNF-α release [112]. The activation of α7 nAchR in macrophages increased also the expression of miR 2055b that mediates the cholinergic anti-inflammatory activity by targeting HMGB1, suggesting that it is also a potential therapeutic target for the treatment of inflammatory diseases [113].

miR-132 is the miRNA that targets AChE in both the brain and the periphery and attenuates the inflammation. In alveolar macrophages, miR-132 inhibits LPS-induced inflammation by enhancing the cholinergic anti-inflammatory pathway and reducing the AChE level. In EAE, miR-132 is downregulated and associated with the severity of EAE, and the activation of miR-132 has an anti-inflammatory effect and mediates attenuation of EAE [114]. miRNA-199a and miR-186 suppress cholinesterases to increase cholinergic signaling, resulting in decreased expression of pro-inflammatory cytokines [109].

## 4. Genetic Polymorphisms of Cholinergic Components

Other than SNPs in non-coding regions of cholinergic genes that have systemic effects and increase the risk of diseases, several variants of genes encoding BChE and AChE have also been largely investigated in relation to the onset of Alzheimer’s disease [115,116], and in relation to altered levels of their enzymatic activity [117]. Nineteen single nucleotide polymorphisms (SNPs) were identified for AChE, and only rs1799806 on exon 6, a functional intronic C/T substitution, was associated with activity changes. The AChE activity of the homozygote Pro/Pro genotype was significantly lower than Arg/Arg genotypes. The presence of these variants is controversially considered a risk factor for Alzheimer’s disease, as well as other conditions such as stroke, Parkinson’s disease, and related dementia [118]. The important study of Simchovitz et al. predicted that SNPs that change miR binding to the 3′-UTR of their target genes may start a domino-like cascade with different regulation and highlighted the dual paradigm of “single miR/many targets” and “single target/many miRs” [119]. The SNP in the 3′-UTR of AChE diminishes the miR-608 binding and regulatory effect, causing both significant elevation of brain AChE activity and down-regulation of other miR-608 targets attributed to “unemployed” miR-608 molecules as the intercellular adhesion molecule CD44 expressed on lymphocytes and leukocytes and the inflammatory-promoting IL6, explaining why, in the presence of SNP, inflammatory markers are only slightly increased despite the elevated levels of AChE, which could limit the cholinergic anti-inflammatory pathway [120]. miR-125b that regulates the IL-6 receptor and miR-608 that regulates IL-6 interact with ACHE gene 3′ UTR variants rs17228602 and rs17228616, with a synergistic anti-inflammatory effect of the two miRs and an amplified inflammatory phenotype in co-carriers of the two SNPs [120,121].

Genetic polymorphisms of CHRNA7 may be a potential marker of dementia, useful to identify a high risk or responder individuals. In fact, CHRNA7 SNPs (rs1514246, rs2337506, rs8027814) seem to possess protective factors in different forms of dementia including AD [122]. SNPs of the *CHRM2* and *CHRM3* genes were evaluated in relation to AD risk and late onset of AD, evidencing weak effects [123].

Contrasting results on the association between the *CHAT* gene polymorphism and increased risk of AD were reported [124,125,126].

## 5. Therapeutic Opportunities

Thinking on the heterogeneous expression of the cholinergic system components in both neuronal and non-neuronal cells, and on the relationship between their dis-regulated expression and function with several diseases, preclinical research utilizing advances in molecular genetics provides a rationale for studyng novel diagnostic and therapeutic approaches in inflammatory and diseases, improving our understanding of the neuronal regulation og immunity and neurodegeneration. 

The manipulation of cholinergic parameters, such as the activities of the ACh hydrolyzing enzymes (specifically selective for AChE or BChE), shows a great importance in the improvement of ACh availability in the treatment of several neuropathologies in which the inflammatory state may be modulated by ACh released both from parasympathetic and immune systems [127,128]. Treatment with AChE inhibitors to inhibit brain AChE reduces the production of IL-1β in both the hippocampus and blood, showing that cholinergic improvement suppresses both central and peripheral inflammation via α7 nAChR, suggesting that the brain’s cholinergic networks communicate with the anti-inflammatory pathway thereby suppressing peripheral inflammation and play a critical role in the protection against the development of neurodegenerative diseases [129].

For this reason, chemical and molecular research combined with animal models, the use of selective muscarinic and nicotinic agonists/antagonists, and AChE/BChE inhibitors and the CholinomiRs regulation may have the potential to identify new avenues to alleviate the burden of many neuronal-immune-mediated diseases.

## 6. Conclusions

The cholinergic network modulates various cellular functions such as neurotransmission, the microglial phenotype in the context of a wider range of neurological disorders, and can influence immune reactions and cells of the peripheral immune system, specifically macrophages and T cells. ACh produced by T cells in a healthy organism plays a key role in the anti-inflammatory pathway (inflammatory reflex). Furthermore, the autocrine cholinergic loop represents an important regulatory mechanism during the maturation, activation, and proliferation of T cells.

Great attention must be given to the well-described link between the central nervous system and terminal effector cells defined as the “cholinergic anti-inflammatory pathway”. The fact that α7 nAChR was expressed also in cells of the immune system has often been undervalued, in spite of α7 nAChR stimulation that can result in the reduction of inflammation, leukocytes extravasation, lymphocyte proliferation, and macrophage and microglia activation ameliorating the disease processes of multiple sclerosis, Alzheimer’s, and Parkinson’s disease. Thus, modulation of the anti-inflammatory pathway of the cholinergic system may represent an approach to treat inflammatory neurological diseases.

Dysregulation in function and expression of the “non-neuronal cholinergic system” components were reported to be important factors in the cause and progression of neuroinflammatory diseases [129,130], and current data may already point to suitable molecular targets for new therapeutic strategies.

Discovery of the microRNAs and SNPs related to many components of the cholinergic system, such as the ACh receptors, VAChT and ChAT other than ChEs, together with the growing range of anticholinesterase treatments, there is a need for critical evaluation of established and novel approaches for manipulating cholinergic parameters that could be disease- and treatment-modifying.

## Figures and Tables

**Table 1:** Expression of the cholinergic system components in immune cells.

Cholinergic Components	Lymphocytes	Monocytes	Macrophages
ACh	+ [14,19,20]	+ [14,19]	+ [5]
ChAT	+ [13]	+ [13]	+ [13]
AChE	+ [13]	+ [20]	+ [20]
VAChT	+ [13]	NR	NR
mAChR	+ [21]	+ [21]	+ [21]
nAChR	+ [12,20,22]	+ [13]	+ [13]

“+” mean that the compound is present.
